# Similarities (and Differences) in the Learning Patterns of Single-Word Reading of an Alphabetic Orthography in Monolingual and Bilingual Primary School Children: A Cross-Sectional Study

**DOI:** 10.3390/brainsci16040356

**Published:** 2026-03-26

**Authors:** Giuditta Smith, Elisa Bassoli, Yagmur Ozturk, Emily Arteaga-Garcia, Wanjing Anya Ma, ROAR Developer Consortium, I-ROAR Data Collector Consortium, Jason D. Yeatman, Marilina Mastrogiuseppe, Sendy Caffarra

**Affiliations:** 1 School of Health Sciences, University of East Anglia, Norwich NR4 7TJ, UK; 2 Department of Biomedical, Metabolic, and Neural Sciences, University of Modena and Reggio Emilia, 41125 Modena, Italy; elisa.bassoli2@unimore.it (E.B.);; 3 Department of Humanities, University of Trieste, 34123 Trieste, Italy; yagmur.ozturk@units.it; 4 Graduate School of Education, Stanford University, Stanford, CA 94305, USA; 5 National Research Council of Italy, Institute for Biomedical Research and Innovation, 98122 Messina, Italy

**Keywords:** reading, bilingualism, lexical decision, literacy acquisition, single-word reading, SES, vocabulary

## Abstract

**Highlights:**

**What are the main findings?**

- Monolinguals and bilinguals show similar developmental patterns in decoding.
- A transient bilingual advantage is observed in the early stages of reading acquisition.

**What are the implications of the main findings?**

- Monolingual and bilingual children show similar final outcomes in learning how to read.
- Language-specific educational practices have a major impact on the early stages of reading acquisition.

**Abstract:**

**Background/Objectives:** With growing waves of migration, children speaking a home language different from the language of school literacy have become increasingly common in Western education systems. In this context, understanding and monitoring bilinguals’ reading development is crucial to inform both educational and clinical practices and ensure equitable services. The present study contributes to the literature by investigating learning patterns in single-word reading across primary school grades. Monolingual and bilingual children learning to read in an alphabetic orthography were examined. **Methods:** The sample consisted of 565 typically developing monolingual and bilingual primary school children from grades 1–5 (bilinguals = 162). Participants completed a computerised Lexical Decision task (LDT) recording accuracy and response times, and standardised tests of reading and cognition. A parental questionnaire was used to gather socio-demographic and linguistic information. **Results:** Response bias-corrected accuracy rates in the LDT revealed an increase in sensitivity across school years after correcting for potential confounds (SES, vocabulary, nonverbal intelligence). No significant effect of bilingualism was observed. Response times for correct responses also decreased consistently across grades after controlling for the same confounds. Although no significant main effect of bilingualism emerged, an interaction with grade revealed a greater decrease in response times for second-grade bilinguals compared to monolingual peers. **Conclusions:** Monolingual and bilingual children showed comparable sensitivity rates and reading times, suggesting similar decoding skill acquisition. However, an earlier decrease in response times for bilinguals points to a facilitatory effect in the early stages of reading development, consistent with a bilingual advantage during skill learning.

## 1. Introduction

Given the increasing flux of migration, multilingualism has become a widespread phenomenon in Western societies and, consequently, in Western schools. Italian families with at least one family member with a citizenship other than Italian represent about 10% of families residing in the country [1], with 11% of children attending Italian schools having a different citizenship [2] (We use the word “citizenship” to align with the available census data. Since children born in Italy to parents/mother of a different nationality do not automatically obtain Italian citizenship at birth (which is obtained via ius sanguinis), this statistic roughly mirrors the number of children of non-Italian descent). These children typically experience exposure to a language at home (their home or heritage language) that is different from the societal language and the language of education, making them bilingual (adopting a broad definition of bilingualism [3,4,5]). Typically, bilingual children will enter the educational system having their heritage language as their dominant (or only) language and will only experience a shift in dominance to either a balanced proficiency or a more prominent proficiency in the societal language throughout the years [6]. When they enter the school system, they are often less proficient in the language of education compared to their monolingual peers, and show greater variability determined by individual differences in language exposure [7,8,9,10,11]. In this paper, we will be addressing the question of how bilingualism influences literacy learning patterns, particularly focusing on the acquisition of decoding of an alphabetic language across primary school.

Reading is a complex human activity that requires the integration of various skills, from ocular movements to phonological and orthographic abilities, as well as semantic integration. Decoding written alphabetic text relies on the ability to compare and connect visual (grapheme) and auditory (phoneme) representations and access their meaning. Models of reading (including the dual route cascaded (DRC) model [12], the connectionist dual process model (CDP ) [13], and the connectionist triangle model [14]) typically divide these operations into different decoding strategies: a serial process of single grapheme-to-phoneme conversion (GPC) (the ‘sublexical’ route in Coltheart and colleagues’ model [12]), and a process of parallel whole-word retrieval for words that have a representation in the orthographic lexicon (the ‘lexical’ route in DRC). While the two mechanisms operate in tandem, the sublexical route is essential for reading nonwords or novel words that have no semantic representation in the mental lexicon, and the lexical route is for quickly reading known words. In learning to read alphabetic orthographies such as Italian, children are taught GPC reading and initially apply it indistinctively to known and unknown words, while, as their orthographic lexicon grows, they increasingly recognise words more quickly through the lexical route [15,16,17]. These stages of development can be thought of as roughly corresponding to the pace of school grade teaching: in the first two years of school, initial GPC reading strategies are taught; between the second and third year, children increase speed and automaticity, relying on lexical access more and more [18].

Monolingual reading processes and outcomes have been widely studied, particularly in relation to prerequisites of reading that are associated with better literacy outcomes in beginner readers, such as rapid naming (RAN) and phonological awareness [19,20,21,22,23]. On the other hand, the literature on the developmental reading trajectories of bilingual learners is quite limited. Reading comprehension abilities have been proposed to be especially affected in bilingual speakers [24,25,26]. This effect has been particularly investigated in English, where measures of English language proficiency such as receptive vocabulary, morphological awareness, and oral language proficiency have been consistently found to predict present and future reading proficiency measures [27,28,29]. Importantly, differences in reading comprehension have been proposed not to be a result of poorer decoding abilities, but rather of overall weaker language proficiency [26,30,31]. In fact, similarities in decoding abilities in monolingual and bilingual children have been reported. In a study on Dutch fourth graders [32], early L2 speakers of the language were even better decoders than monolingual speakers in words and nonwords. In the longitudinal studies of Leasaux and colleagues [33] on bilingual English speakers and Nakamoto et al. [34] on English-Spanish bilinguals, decoding scores showed very comparable decoding skills compared to their monolingual peers (or normative data in the case of [34]) throughout the data collection period. If disadvantages in decoding are found, these are better explained by external factors such as socioeconomic status (SES), age of acquisition, and exposure to the language of education, rather than by bilingualism itself [31,35,36,37].

Another phenomenon that can impact bilingual reading acquisition is related to cross-linguistic transfer effects in cases of bilingual literacy. Studies focusing on the effects of cross-linguistic transfer in multi-literate bilinguals have explored how regularity differences in grapheme-to-phoneme mapping (i.e., a language’s orthographic depth) could intervene in reading strategies. Building on the literature identifying different reading strategies for different languages depending on the “grain size” of the language (i.e., minimal orthographic unit to be mapped onto phonology, [38,39]), theories of bilingual literacy propose that cross-linguistic transfer effects can appear depending on whether the two orthographies have similar or different orthographic depth. For instance, facilitatory transfer effects on reading acquisition can be observed in the case of two relatively transparent orthographies (such as Italian and Finnish) as compared to transparent–shallow pairs (Italian–English) (Grain Size Accommodation Hypothesis, GSA [40]).

Another factor that has been extensively discussed to have a facilitatory effect across language tasks for bilingual speakers is enhanced domain-general abilities (a phenomenon referred to as “bilingual advantage”). This effect is typically explained as the consequence of a consistent use of executive functions that bilingual individuals make during language monitoring and language switching, which would strengthen cognitive processes involving skills such as planning, attention, working memory, and the ability to switch between tasks (e.g., [41,42,43]), and it can be influenced by language and cultural characteristics [44]. The magnitude and specificity of these enhanced executive functions have been largely debated for decades (see [45,46] for a review of the literature). In relation to learning trajectories in particular, the question becomes whether domain-general executive functions are actively employed by bilingual children in a constant manner, or whether they operate within a time window. Domain-generality hypotheses build on the idea that bilingual language use engages cognitive functions any time the bilingual speaker uses one of their languages [41,47,48,49], as the non-target language needs to be actively inhibited by top-down cognitive processes. On the other hand, the skill-learning account applied to bilingualism [50,51,52] builds on the belief that cognition is essentially adaptive, predicting that top-down cognitive processes are highly employed during the problem-solving phase, and are substituted by automatic processes when the task is consolidated in long-term memory and automatised. Skill-learning theories, therefore, add a temporal dimension to the bilingual advantage. When applied to development, the predictions of these accounts are contrasting: while domain-generality accounts predict the bilingual advantage to be constant, i.e., not affected by age, skill-learning accounts predict a larger advantage for younger compared to older children (due to a larger employment of executive functions in the early stages of learning, for a comprehensive review see [53]). While mostly investigated around the advantage in language switching performance, we believe this debate can also be applied to reading, particularly in evaluating whether any facilitatory effect of bilingualism in decoding emerges, persists, or is confined to the early stages of learning.

In light of these considerations, the present study investigates learning patterns in bilingual and monolingual children’s acquisition of decoding. Firstly, we aim to contribute to the discussion on whether bilingualism has any effect on learning to decode an alphabetic language, after controlling for potential confounds such as SES, language exposure, nonverbal intelligence, and vocabulary. Our work will focus on the acquisition of an alphabetic, transparent language, adopting a cross-sectional methodology that embraces all primary school years. The relevance of this aim is both theoretical, since few studies investigating the process of single-word reading development from the first to the last year of primary school exist, and educational. In fact, the number of pupils learning to read in Italian schools while having a different language at home is ever-increasing, and research can inform the educational sector to maximise inclusion and participation. Secondly, we focus on the duration of any effect of bilingualism across school years, aiming to address the question of whether the effect is constant or limited to a specific phase of development. If early phases of learning to decode reflect differences in domain-general executive functions (as predicted by skill-learning accounts, [50,51,52], then we expect group differences between bilinguals and monolinguals to be most evident in the earlier years and to diminish with time and increased automatization. In other words, any bilingual effect in decoding accuracy and/or speed should be time-limited, emerging earlier and reducing or disappearing by the later phases of reading acquisition. If, instead, the difference due to bilingualism is constant over time, then differences should persist across primary school, rather than narrowing with age.

## 2. Materials and Methods

### 2.1. Participants

Children were recruited from six primary schools in Modena and eight in Trieste, both located in northern Italy. Italian primary education typically spans ages 6 to 11 and is structured in five grades. Participants included in the study were part of a larger data collection validating the lexical decision task (LDT) described in Section 2.3. For the present study, sample selection started from the 1515 children who completed at least 50% of the LDT task (described in Section 2.3) and whose performance was not indicative of a random response pattern (mean age = 8.82; age range = 6.2–11.5, SD = 1.29). Participants with no certified disability, neurodevelopmental disorder—including dyslexia—or reading difficulty, and whose parents completed the whole family questionnaire described in Section 2.3, were retained, leading to a final sample of 565 children (318 females; mean age = 8.7 years, range = 6.2–11.2, SD = 1.28) across all five primary school grades. Of these, 162 were bilingual (28.6%).

**Figure 1:**
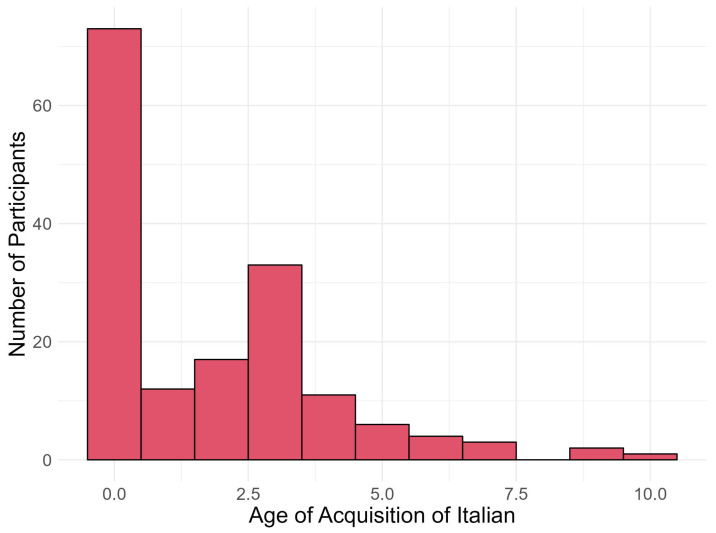
Barplot representing the age of acquisition of Italian in the bilingual sample (N = 162).

The bilingual children in the sample spoke a variety of languages other than Italian. The most common were Arabic (N = 31), English (28), Albanian (27), Romanian (19), and Tagalog (10). The majority of bilingual children were born in Italy (N = 140, 86%) and had attended all three years of nursery school in Italy (N = 119, 73%), having early exposure to Italian, as illustrated in Figure 1. Regarding exposure to languages other than Italian, most bilingual children were exposed to their home language within the family context (N = 136, 84%), and about half were learning/had learnt to read in another language (N = 74, 46%).

### 2.2. Background Tasks

A questionnaire was developed to gather information on the participants’ clinical profile, linguistic background, and socio-economic status. The questionnaire was developed on Google Forms and was available in Italian and English. The English translation was supervised by a first-language speaker. Printed copies were provided to the schools to distribute to parents who did not possess or did not regularly have access to computers.

Participants were administered standardised tasks measuring reasoning and vocabulary. For nonverbal reasoning, we adopted the Raven’s Coloured Progressive Matrices (CPM) [54]. In the task, participants are asked to complete a series of coloured puzzles, for a total of 36 items. Each puzzle is missing one piece, and the participant must select one of six alternatives. For vocabulary, we administered the Boston Naming Test (BNT) [55], where participants are shown a series of images and are asked to name them in Italian, for a total of 60 items. Table 1 presents the distribution of participants across grades and their average scores on the standardised measures of nonverbal reasoning (CPM) and vocabulary (BNT).

### 2.3. Experimental Tasks

An LDT was adopted to investigate the acquisition of single-word reading [56], in which participants are asked to decide whether a string of letters is a real word or not. LDTs have been utilised successfully in the literature to investigate decoding skills. The underlying mechanisms of the high correlation between lexical decision tasks and standardized reading scores are still not fully understood. However, potential explanations come from findings in cognitive psychology and neuroscience. In cognitive psychology, research studies suggest that some key cognitive processes at play when participants make a dual forced choice lexical decision are also at play during visual word recognition [57,58,59,60]. Moreover, recent neuroscientific models postulate that a crucial stage of natural reading is lexical categorization [57,58,61,62,63]. The task is the Italian adaptation of the LDT of the Rapid Online Assessment of Reading (ROAR) suite [64], a platform containing several online automated tests functioning as screening tools for reading abilities and reading prerequisites. The English and Spanish versions of the task have been shown to have high validity and reliability compared to available standardized tasks to screen decoding abilities [64,65,66], as well as the Italian adaptation employed here [56].

**Figure 2:**
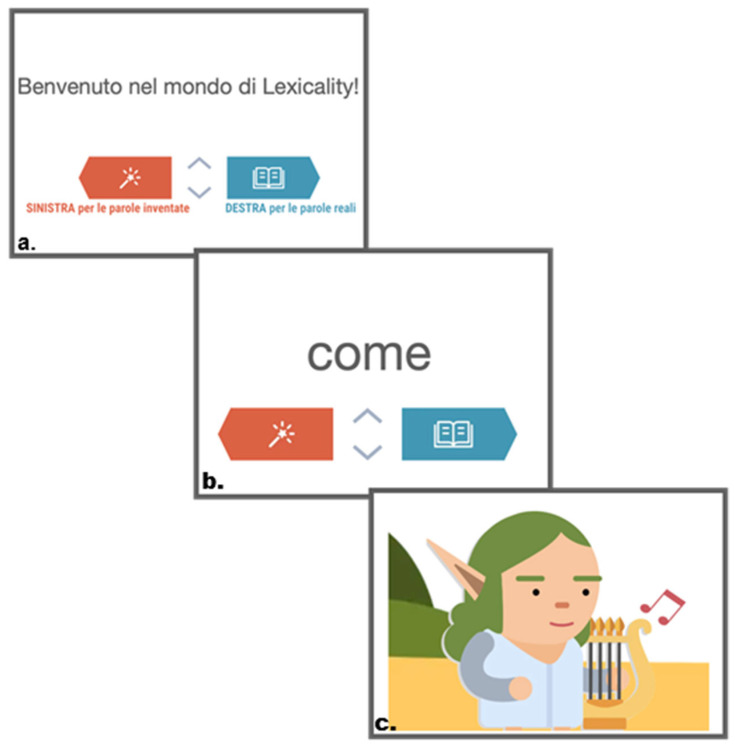
An example of the LDT screens presented to the child: (**a**) instructions (Welcome to Lexicality! Left button is for made-up words, right button is for real words); (**b**) an example of a real-word stimulus (*come*, “how”); (**c**) one of the characters of the gamified story.

The LDT was self-administered online from a tablet or computer. A string of letters appeared on the screen, disappearing after 350 ms with no timeout. All stimuli were presented in lowercase, which is taught in Italian schools from the later stages of the first grade of primary school. Participants were instructed by a narrating voice to press a button to choose whether the string of letters is meaningful (a word) or not (a pseudoword). An acoustic feedback signalled the accuracy of the selection with a trill (accurate) or a thud (not accurate). Each participant saw a total of 170 words (50% words, 50% pseudowords) randomly selected from a pool of 286 items (143 word–pseudoword pairs). Examples of pseudowords are: *accivista* and *pedre*. The task was gamified to make it more engaging for children in the form of a story with different fantasy characters. Figure 2 shows an example of the screen as presented to the children.

### 2.4. Procedure

Participants were tested at school during regular school hours. Up to four children at a time were accompanied in a quiet room and were tested individually. This testing phase included three parts: (a) a tablet-based session, during which the children independently completed the ROAR task while wearing headphones and sitting apart from the rest of the group; (b) a one-on-one session with the researcher, during which standardised tests were administered, and (c) class administration of the reasoning task (CPM). This took place on a different day compared to (a) and (b) during regular school hours and was carried out by either the teachers or the researchers involved in the study. The individual sessions ((a) and (b)) took approximately 30–40 min. For the I-ROAR word, children were instructed to keep the tablet in the same position and respond as quickly and accurately as possible.

### 2.5. Data Analysis

In this study, we examined the differences in learning curves on a lexical decision task in monolingual and bilingual children, while controlling for potential confounds of SES, vocabulary size, nonverbal intelligence, and bilingual measures of exposure. SES was calculated using the Four Factor Index of Social Status, based on each parent’s education and occupation [67]. Parental educational level was assigned a score from 1 to 7, and occupational level a score from 1 to 9. SES scores for fathers and mothers were computed using the formula (educational level × 3) + (occupation × 5), and the child’s SES was derived as the mean of the two parental scores. Bilingual measures were age of acquisition of Italian (continuous variable, with 0 = since birth); years of nursery education in Italy (continuous variable); whether a parent spoke a language other than Italian at home (home language use, categorical variable); whether the child was mono- or biliterate (categorical variable: only Italian, or Italian + other languages). To test for collinearity issues, the variance inflation factor (VIF) was calculated, and all predictors with VIF < 2.5 were retained (conservative cut-off for a risk of instability of beta coefficients [68]).

To investigate bias-corrected accuracy, we computed *d’* based on Signal Detection Theory [69]. *d’* is a measure of sensitivity, i.e., the participant’s ability to distinguish real words from pseudowords independently of response bias. Higher *d’* values indicate better discrimination. For each participant, we first calculated the number of hits (correctly identifying real words), misses (failing to identify real words), false alarms (incorrectly identifying pseudowords as real), and correct rejections (correctly identifying pseudowords as pseudo). To avoid infinite z-scores when hit or false alarm rates were 0 or 1, we applied the Hautus log-linear correction, adding 0.5 to each count and 1 to each denominator. Corrected hit and false alarm rates were then transformed into z-scores to compute *d’*. Finally, to examine predictors of lexical discrimination ability, a multiple linear regression model was fitted with *d’* as the dependent variable, with fixed factors of language status and grade, as well as their interaction, and covariates for linguistic and cognitive factors to control for potential confounds (CPM, BNT, SES, age of acquisition, years of nursery, home language use, reading in other languages). To test the significance of fixed effects, we applied a Type II ANOVA using the car package 3.1.2 [70]. Effect sizes are reported as partial eta squared (ηp^2^) for all omnibus tests.

For each subject and stimulus type (word, pseudoword), only response times for correct responses of trials within 2 SD of the mean RT were retained. A linear mixed effect model was fitted using the lme4 package 1.1.33 in R to log-transformed response time data on correct trials [71]. The model included fixed factors of bilingualism and grade, and covariates of linguistic and cognitive factors to control for potential confounds. By-subject and by-item random intercepts were included. To test the significance of fixed effects, we applied a Type II ANOVA using the car’ package. Effect sizes for fixed effects are reported as ηp^2^. Model fit for the mixed model is described using marginal and conditional R^2^.

## 3. Results

### 3.1. Sensitivity

**Figure 3:**
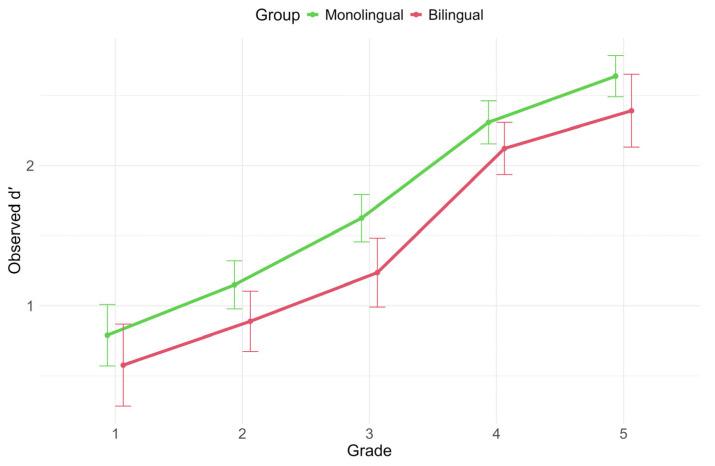
Observed *d’* values across grades, divided by group.

Observed results per grade and language group are shown in Figure 3. The model (multiple R^2^ = 0.54, adjusted R^2^ = 0.52) reveals a significant main effect of grade on *d’* (*F*(4, 548) = 56.75, *p* < 0.001, ηp^2^ = 0.29), indicating that sensitivity varied across grade levels. In contrast, neither language status (*F*(1, 548) = 0.17, *p* = 0.683, ηp^2^ < 0.001) nor the interaction between language status and grade (*F*(4, 548) = 0.25, *p* = 0.9, ηp^2^ < 0.001) accounts for *d’* changes after controlling for potential confounding variables.

Vocabulary size, as measured by the BNT score, is positively associated with *d’*, meaning a richer vocabulary leads to higher sensitivity (*F*(1, 548) = 41.25, *p* < 0.001, ηp^2^ = 0.07). The same effect and direction were reported for CPM (*F*(1, 548) = 17.93, *p* < 0.001, ηp^2^ = 0.03), indicating that higher nonverbal reasoning abilities produce higher levels of sensitivity. There is also a trend for SES (*F*(1, 548) = 3.67, *p* = 0.056, ηp^2^ < 0.001), indicating higher sensitivity in high-SES children.

Of the bilingual-specific variables, only the effect of home language exposure is significant (*F*(1, 548) = 5.65, *p* = 0.018, ηp^2^ = 0.01), showing that higher exposure to Italian at home is associated with higher sensitivity.

### 3.2. Response Times

**Figure 4:**
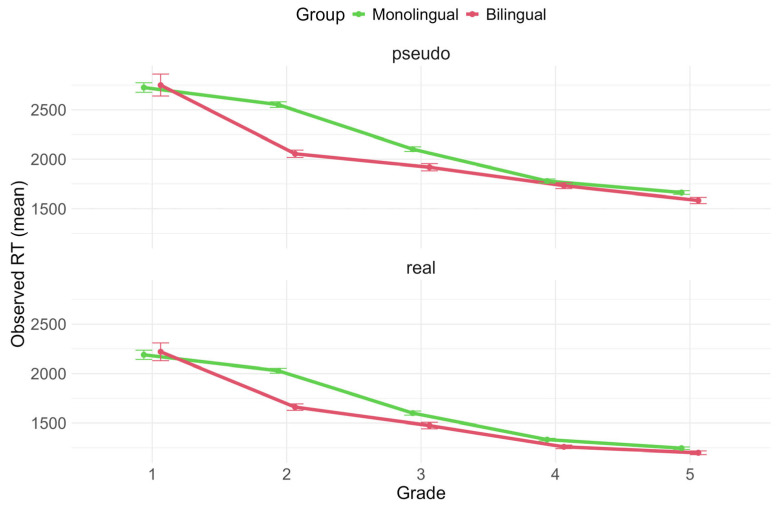
Observed RT values for correct responses across grades, divided by group and stimulus type.

Response times for correct responses visualised by school grade are shown in Figure 4. The model (conditional R^2^ = 0.45, marginal R^2^ = 0.2) reveals a significant main effect of grade (*F*(4, 545.74) = 55.84, *p* < 0.001, ηp^2^ = 0.29), where response times progressively become faster over time. There is also a main effect of stimulus type (*F*(1, 272.43) = 984.97, *p* < 0.001, ηp^2^ = 0.21), with words being processed faster than pseudowords.

No simple main effect of language status emerges in this case. However, a significant interaction between language status and grade is reported (*F*(4, 546.16) = 3.70, *p* = 0.006, ηp^2^ = 0.03). Post hoc FDR-corrected statistics for the two-way interaction reveal that the effect of language status is only significant in second grade (estimated difference = −438 ms, 95% CI [75, 801], z = 3.96, *p* = 0.0002), meaning that second-grade bilinguals have faster response times than second-grade monolinguals after correcting for vocabulary, nonverbal intelligence, family SES, and bilingual variables. This result is further confirmed by a follow-up analysis on a subset group of second graders (N = 72, of which 36 were monolingual and 36 were bilingual) matched for CPM scores and SES (*F*(1, 64.94) = 4.21, *p* = 0.044, ηp^2^ = 0.06).

Looking at grade progression in the two groups, bilinguals in second grade differ significantly from bilinguals in first grade (estimated difference = −545 ms, 95% CI [73, 1017], *z* = 3.76, *p* = 0.0003), while monolinguals show a similar significant difference a year later, between second and third grade (estimated difference = −448 ms, 95% CI [217, 680], *z* = 6.32, *p* = 0.0001), confirming an earlier reduction in response times for bilinguals compared to monolinguals after correcting for potential confounds. Monolingual and bilingual children’s performance does not differ in fourth and fifth grade (*zs* < 1.5, *ps* > 0.1). No significant effects of the confounding variables were reported.

## 4. Discussion

The present study investigated the development of decoding skills in bilingual and monolingual children learning to read in an alphabetic, transparent orthography (Italian). Using a cross-sectional design, learning patterns of sensitivity and response times to an LDT were investigated from the first year in which children learn to read to the end of primary school (from 6 to 11 years of age). Two main questions guided the study: first, whether bilingualism exerts facilitatory effects in decoding acquisition above and beyond linguistic and contextual factors that are strongly associated with bilingual differences (namely, vocabulary, SES, and age of acquisition); and second, whether any effect of bilingualism is stable across development or restricted to a specific phase of learning. We acknowledge the following limitations of the study: first, while the cross-sectional design adopted gives us insight into learning patterns and processes, it cannot directly inform us on learning trajectories, for which a longitudinal design is necessary. Secondly, the bilingual children who took part in the study are mostly described as early bilinguals, with early exposure to Italian. Therefore, we cannot draw conclusions that generalise to all bilinguals, including late bilinguals. Lastly, although we find no general effect of biliteracy, at present, we do not investigate in depth potential transfer effects for the biliterate children in the sample.

Looking at overall progression across school grades, decoding performance showed a clear increase in sensitivity and an inverse trend for response times. Children became both more accurate and faster in determining whether a stimulus was a word or a pseudoword as school years progressed. This result is consistent with models of reading in children positing a shift from GPC decoding to more automatised and faster word recognition with increasing experience and exposure [15,16], as well as with longitudinal and cross-grade evidence showing that once a sufficient level of decoding accuracy is reached, subsequent development is characterised by increasing speed and efficiency (e.g., [72,73]). Crucially, we found these developmental patterns of decoding skills to be overall similar in monolingual and bilingual learners once factors that constitute sources of variance in bilinguals are controlled for. In fact, linguistic background was not found as a main effect in either response times or sensitivity, but the latter was modulated by both vocabulary size, nonverbal cognition, and SES. This finding contributes to the mixed results previously found in the literature, supporting accounts that do not posit systematically worse decoding abilities in bilingual students in word-level reading development, but rather comparable growth patterns modulated by other factors, most notably vocabulary knowledge, language exposure, and SES [31,35,36,37,74,75]. Future studies adopting a longitudinal design are needed to further strengthen the generalizability of this result to decoding developmental trajectories.

The association between decoding accuracy and vocabulary size, as well as socioeconomic status, is in line with evidence showing that SES-related differences in reading outcomes are largely mediated by variation in lexical knowledge, rather than decoding mechanisms per se [72,76]. For bilingual-specific variables, only home language exposure showed a modest but significant association with decoding sensitivity in our data, such that greater exposure to Italian at home was linked to higher sensitivity. This result is consistent with the previous literature, where greater exposure to the dominant language at home is linked to higher literacy outcomes in that language. Language exposure seems to affect multiple key skills of reading, such as visual word recognition, vocabulary, and text comprehension [77,78]. Although our focus here was on decoding, additional studies can provide a more nuanced overview of which subskills of reading are majorly affected by the bilingual experience, as well as a more nuanced conceptualization of home exposure as a cumulative, continuous variable [79,80].

Another strong predictor of word-reading outcomes in bilingual speakers found in the literature is earlier exposure to the language of schooling, with earlier exposure leading to stronger outcomes [31,81]. In the present study, the effect was not replicated. However, this is likely due to the low variability of this measure in our sample. In fact, as noted in the description of the bilingual participants, most of them were early bilinguals, having a similar early age of exposure to Italian and having been in the Italian pre-schooling system for 2–3 years before the start of primary school. More variability in language dominance and exposure will allow us to test for decoding differences in both sensitivity and response times. It is also worth mentioning that we do not find an effect related to whether the children are mono- or biliterate (i.e., the categorical variable of biliteracy was not significant for either sensitivity or response times). Future studies should explore cross-linguistic transfer given by specific orthographic pairs to expand the debate around the GSA with developmental data and examine whether the present findings generalize to bilingual populations in non-European contexts, where greater variability in orthographic systems and sociolinguistic environments may lead to different developmental trajectories.

A sensitive measure, such as response times, allowed us to identify a subtle difference in their learning patterns: while the main effect of language status was not significant, an interaction between language status and grade was significant for second graders, revealing that bilingual pupils in second grade were faster than their monolingual peers in single word recognition. This earlier drop in response times appears during a phase of reading acquisition where fluency and processing speed are consolidating. This stage marks a transition toward increasingly automatised word recognition [18], during which performance remains particularly sensitive to individual differences [72]. We interpret this drop at the transition stage in bilingual speakers as directly relevant to the debate over whether any potential facilitatory effect is constant over time or limited to a specific learning window. Specifically, by providing new insights from the domain of decoding acquisition, this work helps disentangle domain-general accounts [48,82] from skill-learning accounts of bilingualism [50,51,53]. Since we only find a facilitatory effect in the early phase of reading development (which is coherent with the onset of the automatization process), our data are in line with skill-learning accounts suggesting a transient efficiency effect during skill acquisition. This is compatible with a potential facilitatory effect of bilingualism during an intermediate stage of skill consolidation, followed by convergence across groups as decoding becomes increasingly automatised.

## 5. Conclusions

The present study investigated whether bilingual and monolingual children follow a similar developmental pattern in learning to decode written words in an alphabetic orthography. Overall, decoding accuracy and speed improved with grade in both groups, supporting the view that, when accounting for vocabulary, SES, and bilingual variables, word-level reading developmental patterns are largely shared across language backgrounds. This result has educational and clinical implications, particularly in the screening of decoding abilities throughout the school years. This practice is essential for monitoring individual developmental trajectories and for early detection of learners lagging behind their peers. Bilingual children with early exposure to Italian show a pattern of decoding development similar to that of monolingual peers, with a facilitatory effect in the early stages of reading acquisition.

Crucially, the only group difference observed was a transient advantage in decoding speed for bilingual children in the second grade. The second-grade-specific effect may suggest that executive control mechanisms support processing efficiency during an intermediate phase of decoding consolidation. However, their role diminishes as reading becomes increasingly automated. Future longitudinal studies should further confirm this finding. From an educational perspective, these findings highlight the importance of focusing on children’s language experience, vocabulary development, and stage of reading acquisition. Instructional practices should therefore prioritize supporting decoding fluency during transitional phases of learning—particularly in the early primary years—while recognizing that bilingual and monolingual learners ultimately converge in word-level reading outcomes.

## Figures and Tables

**Table 1:** Descriptive statistics for the participants divided by grade: Sample size (N), mean age in years (with SDs in parentheses); mean raw scores (and SDs) and percentiles on reasoning (CPM); mean raw scores (and SDs) and z-scores on vocabulary size (BNT).

Grade	N (F, Bil)	Age	CPM Score	CPM Percentile	BNT Score	BNT z-Score
1	54 (28, 15)	6.9 (0.29)	29.1 (4.81)	72.9 (30.61)	31.2 (8.6)	0.21 (1.22)
2	138 (81, 37)	7.5 (0.33)	30.3 (4.3)	82.9 (24.06)	32.5 (9.66)	0.13 (1.36)
3	116 (58, 35)	8.4 (0.32)	32.1 (3.51)	85.2 (19.29)	33.6 (8.83)	−0.6 (1.55)
4	120 (75, 41)	9.4 (0.32)	33.4 (3.09)	85.1 (18.95)	37.4 (8.64)	−0.66 (1.53)
5	137 (76, 34)	10.4 (0.3)	33.5 (2.89)	77 (24.51)	42.2 (8.36)	−0.35 (1.34)

## Data Availability

The original data presented in the study are openly available in Zenodo at https://zenodo.org/records/18874035 (accessed on 22 March 2026).

## Abbreviations

The following abbreviations are used in this manuscript:

DRC	Dual Route Cascade model
CDP	Connectionist Dual Process model
GPC	Grapheme-to-Phoneme Conversion
GSA	Grain Size Accommodation
LDT	Lexical Decision Task
